# Assessment of the embolization effect of temperature-sensitive p(N-isopropylacrylamide-co-butyl methylacrylate) nanogels in the rabbit renal artery by CT perfusion and confirmed by macroscopic examination

**DOI:** 10.1038/s41598-021-84372-w

**Published:** 2021-03-01

**Authors:** Zhen Zhang, Chunyuan Cen, Kun Qian, Han Li, Xin Zhang, Hongsen Zhang, Guina Ma, Yan Chen, Nanchuan Jiang, Chuansheng Zheng, Yanbing Zhao, Ping Han

**Affiliations:** 1 Department of Radiology, Union Hospital, Tongji Medical College, Huazhong University of Science and Technology, Wuhan, 430022 China; 2 Department of Radiology, Peking University Shenzhen Hospital, Shenzhen, 518036 China; 3 Hubei Province Key Laboratory of Molecular Imaging, Wuhan, 430022 China; 4 National Engineering Research Center for Nanomedicine, College of Life Science and Technology, Huazhong University of Science and Technology, Wuhan, 430074 China

**Keywords:** X-ray tomography, Nanoparticles

## Abstract

Transcatheter embolization is an important treatment method in clinical therapy, and vascular embolization material plays a key role in embolization. The temperature-sensitive p(N-isopropylacrylamide-co-butyl methylacrylate) (PIB) nanogel is a novel embolic agent. To evaluate the feasibility of the nanogel as a blood vessel embolization agent, we aimed to assess the effect of embolization with PIB nanogels in the rabbit renal artery by non-invasive computed tomography (CT) perfusion, macroscopic and histological examination. Ten healthy adult Japanese rabbits were used to implement RAE of PIB nanogels in their right kidneys. CT perfusion scans were performed pre- and post-treatment at various time-points (1, 4, 8, and 12 weeks). Two rabbits were euthanized and histologically examined at each time-point, and the remaining rabbits were euthanized at 12 weeks after embolization. The RAE efficacy of the nanogels was further confirmed by macroscopic and histological examination. The renal volume and renal blood flow (BF) of the right kidney were significantly decreased post-treatment compared with those pre-treatment (volume: pre, 9278 ± 1736 mm^3^; post 1 week, 5155 ± 979 mm^3^, *P* < 0.0001; post 4 weeks, 3952 ± 846 mm^3^, *P* < 0.0001; post 8 weeks, 3226 ± 556 mm^3^, *P* < 0.0001; post 12 weeks, 2064 ± 507 mm^3^, *P* < 0.0001. BF: pre, 530.81 ± 51.50 ml/min/100 ml; post 1 week, 0 ml/min/100 ml, *P* < 0.0001; post 4 weeks, 0 ml/min/100 ml, *P* < 0.0001; post 8 weeks, 0 ml/min/100 ml, *P* < 0.0001; post 12 weeks, 0 ml/min/100 ml, *P* < 0.0001). No revascularization or collateral circulation was observed on histological examination during this period, and PIB nanogels were dispersed in all levels of the renal arteries. Twelve weeks after embolization, CT perfusion showed no BF in the right renal artery and renal tissue, a finding that was consistent with histological examination showing complete embolization of the right renal artery with a lack of formation of collateral vessels. The effect of embolization on PIB was adequate, with good dispersion and permanency, and could be evaluated by non-invasive and quantitative CT perfusion.

## Introduction

With the rapid development of interventional radiological technology, blood vessel embolism materials (including metal coils, anhydrous ethanol, Lipiodol, onyx, and polyvinyl alcohol (PVA)) have expanded the clinical indications, such as the treatment of postpartum hemorrhage, gastrointestinal bleeding^1–3^, preoperative embolotherapy of tumors^4,5^, and embolization of vascular malformations^6^. However, the clinical application of these materials has some complications, including poor flowability of the solid embolic agent that makes it difficult to uniformly embolize the peripheral vessel. Additionally, solid embolic agents such as spring coils cause obstructive nephropathy^7^. Although liquid embolic agents have good flowability, Lipiodol embolization is often incomplete or totally eradicated by tissue, pulmonary hypertension is often secondary to ethanol embolism^8^, and high concentrations of onyx can result in neurotoxicity^9^. The ideal vascular embolization material has the following characteristics: good dispersibility, radiopacity, biocompatibility and embolism permanency^10,11^. Therefore, it remains a considerable challenge to develop safe and effective liquid embolic materials with a peripheral embolization effect.

Temperature-sensitive nanogels have attracted increasing attention during the last decades because they undergo reversible and rapid volume phase transitions in response to ambient temperature. First, these nanogels have good fluidity in fine catheters because they remain in the liquid state at a low critical solution temperature (LCST). Second, their small size enables them to overcome some biological barriers. Furthermore, a gel forms in situ, conforming to the shape and size of the blood vessels, minimizing trauma to non-target vessels and reducing the use of nanogels. In addition to good dispersibility and permanent embolism, the *p*(*N*-isopropylacrylamide-*co*-butyl methylacrylate) (PIB) nanogels developed by us exhibited a lower inflammatory vascular response, better fluidity, and better operability than PVA-embolized particles and Lipiodol^12,13^. Temperature-sensitive PIB nanogels have been used as novel blood vessel embolic materials to solve the dilemma of peripheral artery embolization and permanent embolization^12–14^. With the progress of preparation technology, the resultant PIB nanogels have become more biocompatible, and the convenience of operation has been markedly improved. Since PIB nanogels have different mechanisms of temperature sensitive artery embolization from other clinical embolic agents, it is difficult to precisely evaluate their performance in renal artery embolization (RAE).

Digital subtraction angiography (DSA) has long been the imaging gold standard in the evaluation, treatment, and follow-up of vascular disorders^15^. Some researchers have attempted to use DSA to assess the long-term effect of embolization^12–14,16^. However, a major drawback of DSA is the invasiveness of vascular puncture. Computed tomography (CT) perfusion imaging is a new, accurate, non-invasive, functional imaging technology that can provide information on hemodynamic changes in organs and tissues^17,18^. One study showed that CT perfusion imaging could provide data concerning renal morphological changes and quantitative renal hemodynamic data^19^. Thus, it is reasonable to use CT perfusion to assess the embolization effect of PIB in the renal artery.

In our previous study^13^, PIB was first developed as a novel temperature-sensitive blood vessel embolic agent to address the dilemma of flowability and embolization in transarterial chemoembolization of hepatocellular carcinoma. This study aimed to observe the feasibility of PIB as a blood vessel embolization agent using CT perfusion imaging, macroscopic and histological examination, thus providing an experimental basis for the clinical application of a novel embolic agent.

## Materials and methods

### Experimental materials

**Figure 1:**
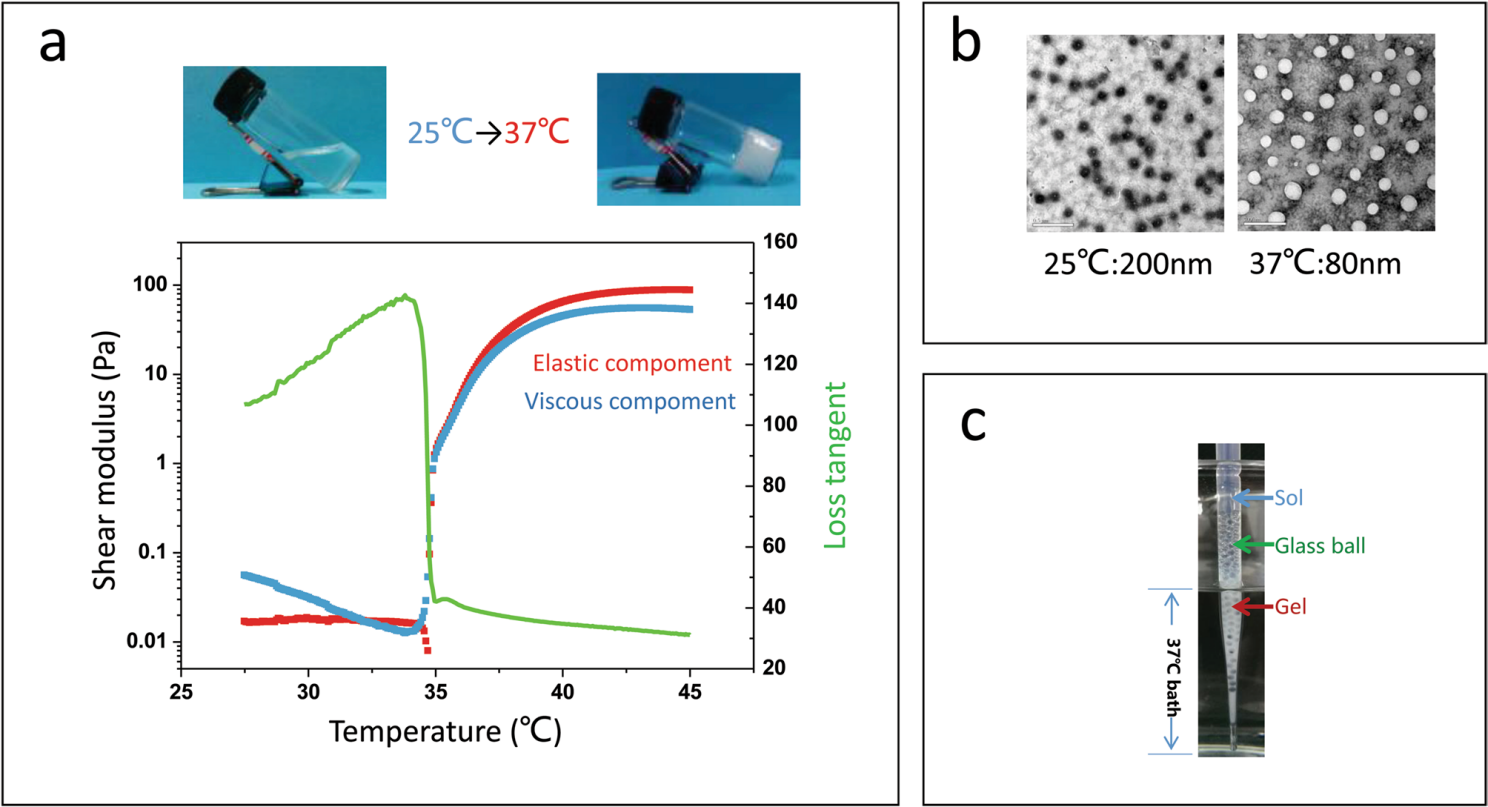
Temperature-sensitive PIB nanogel sol–gel phase‐transition behaviors. (**a**) Temperature modulus testing found that the liquid–solid phase changed at 35 °C. (**b**) TEM images show the diameter of the PIB nanogels at 25 °C and 37 °C. (**c**) PIB nanogels were packed in a 2 mm diameter glass ball tube whose lower segment was immersed in a 37 °C constant temperature water bath; the liquid gel underwent phase transformation for a gel solid below the liquid level.

PIB nanogels, as an embolic agent in this experiment, were provided by the National Engineering Research Center for Nanomedicine, College of Life Science and Technology, Huazhong University of Science and Technology. The PIB nanogel is a new type of temperature-sensitive nanogel that remains in a liquid state at a low temperature and turns into a gelatinous solid at temperatures above its LCST (35 °C). Because it undergoes a transition from a flowable sol phase to a solidified gel phase at its LCST, the PIB nanogel is in a liquid state at a lower temperature (25 °C, compared with 35 °C) and turns into a solid white gel at a higher temperature (37 °C, compared with 35 °C) (Fig. 1).

### Study design

The experiment was designed to assess PIB nanogels and monitor their residue time. The right renal artery of rabbits was embolized with PIB nanogels, and CT perfusion was performed pre- and post-treatment at various time points (1, 4, 8, and 12 weeks). Efficacy was confirmed by macroscopic and histological examination after CT perfusion.

### Animal model preparation

Ten healthy adult Japanese long-eared white rabbits were provided by the experimental animal breeding plant of Tongji Medical College, Huazhong University of Science and Technology. The rabbits were aged 3–5 months, had body weights between 2.5 and 3 kg, and were of either sex. All animal procedures were performed in accordance with the ARRIVE guidelines and National Institutes of Health Guidelines for the Care and Use of Laboratory Animals, and the study was approved by the Institutional Animal Care and Use Committee at Tongji Medical College, Huazhong University of Science and Technology (Certificate no. IACUC-2018–2274).

### Vascular embolization protocol

**Figure 2:**
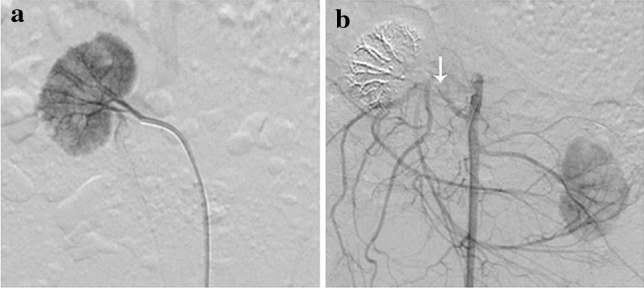
Right renal artery angiography of rabbits. (**a**) Before embolization. (**b**) After embolization. The white arrow indicates that the right renal artery is embolized.

First, the animals were fasted for 12 h before each procedure. Sodium pentobarbital (2.0 wt%, 30 mg/kg) was injected intravenously for anaesthesia. Next, interventional procedures were performed using a DSA unit with strict sterile technique. The right femoral artery was isolated and accessed through an open puncture using an 18-gauge sheath needle. After successful puncture, the needle core was withdrawn and a short guidewire was inserted. Then, the sheath of the puncture needle was withdrawn and a 4F arterial sheath was placed into the femoral artery. Sodium heparin (100 IU/kg) was then administered using a three-way switch. A 4F Cobra catheter was inserted into the right renal artery using a 4F arterial sheath. After injecting a contrast agent (Iopamiron, Bayer HealthCare, 350 mg I/mL; 3 ml/s; a dose of 6 ml per rabbit) into the rabbits, renal arterial angiography was performed. After right renal arterial angiography, the PIB nanogels were injected into the rabbit with an average dose of 2 ml per rabbit via the 4F Cobra catheter (Fig. 2). The distal branches of the renal artery were embolized. Next, the catheter was slightly retracted to embolize the proximal vessels of the renal artery. After injection of the PIB nanogels, the catheter was withdrawn, and the distal femoral artery near the puncture point was ligated.

### Perfusion CT

The parameters of perfusion CT scanning and contrast agent injection are summarized in Table 1. A 320-detector row CT scanner (Aquilion One; Toshiba Medical Systems) was used in the volumetric scan mode. A 22-gauge needle was placed in the ear vein of the rabbit, and sodium pentobarbital (2.0 wt%, 30 mg/kg) was injected intravenously for anesthesia. Next, a nonionic contrast material (Iopamiron; 350 mg I/mL; Bayer HealthCare) was administered via a power injector (Ulrich CT Plus 150; Ulrich Medical). The scan area of the perfusion CT was set to cover at least both kidneys. During the scan, the rabbits were placed in the supine position, and straps were fixed to the rabbit's subcostal abdominal wall to minimize image artifacts caused by respiratory movements. Perfusion was calculated using the maximum slope model (Body Perfusion; Toshiba Medical System), and the results were shown in ml/min per 100 ml. Additionally, color perfusion maps of renal blood flow (BF) were obtained.

**Table 1:** Parameters of perfusion CT.

Parameters	
**Scan parameters**	
Number of detector rows	320
Craniocaudal coverage	160 mm
Collimation	0.5 mm
Tube voltage	80 kVp
Tube current	60 mA
Gantry rotation time	0.5 s
Matrix	512 × 512
Field of view	300 mm × 350 mm
**Contrast agent**	
Iodine concentration	350 mg/mL
Total dose	3 mL
Injection rate	0.5 mL/s
Saline flush	8 mL with 0.5 mL/s
Scan	Every 2 s for 18 s
	Every 3 s for 15 s
	Every 7 s for 35 s

The first scan occurred 6 s after initiating the contrast agent injection.

### Pathology

#### Macroscopic and microscopic pre- and post-RAE treatment

At various time points (1, 4, and 8 weeks), two rabbits were euthanized after CT perfusion was completed, the remaining rabbits were euthanized at 12 weeks, and the kidneys were harvested for general pathological observation. Next, a coronal incision of the kidneys was performed, and a dividing ruler was placed next to the kidneys. The macroscopic presentation at each time point was observed, noting the size, shape, texture, color, cortex, and medullary infarction of the right kidney after the RAE, in addition to observing the presence of any surviving kidney tissue. The post-RAE renal tissues were fixed in 10% formalin for 48 h, dehydrated in graded alcohol, and embedded in paraffin. Then, 4- to 5-μm sections were cut. The tissue samples were then stained with Masson’s trichrome at various time points to evaluate ultrastructural changes and estimate the treatment effects.

### Statistical analysis

Statistical analysis was performed using SPSS 20.0 and Microsoft Excel. All the data were expressed as mean ± standard deviation. Dunnett’s tests were used to assess the differences in the renal volume and BF in the right kidney pre- and post-treatment during the 12 weeks.

## Results

### Dispersion and permanency

**Figure 3:**
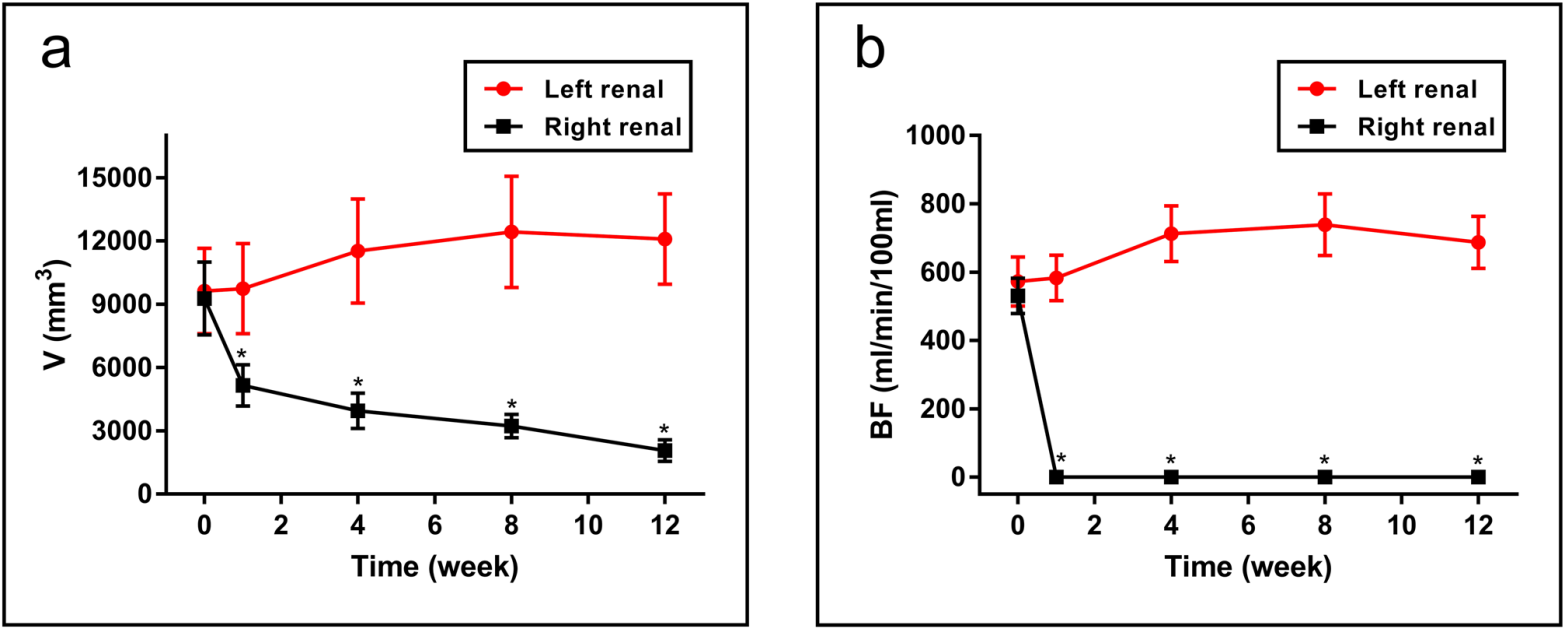
Morphological and hemodynamic changes of the two kidneys of a rabbit at various time points. (**a**) Changes in volume: individual renal volumes were measured using the ellipsoid formula (volume = length × width × thickness × π/6). (**b**) Changes in BF. *P < 0.0001 versus the pre-treatment.

**Figure 4:**
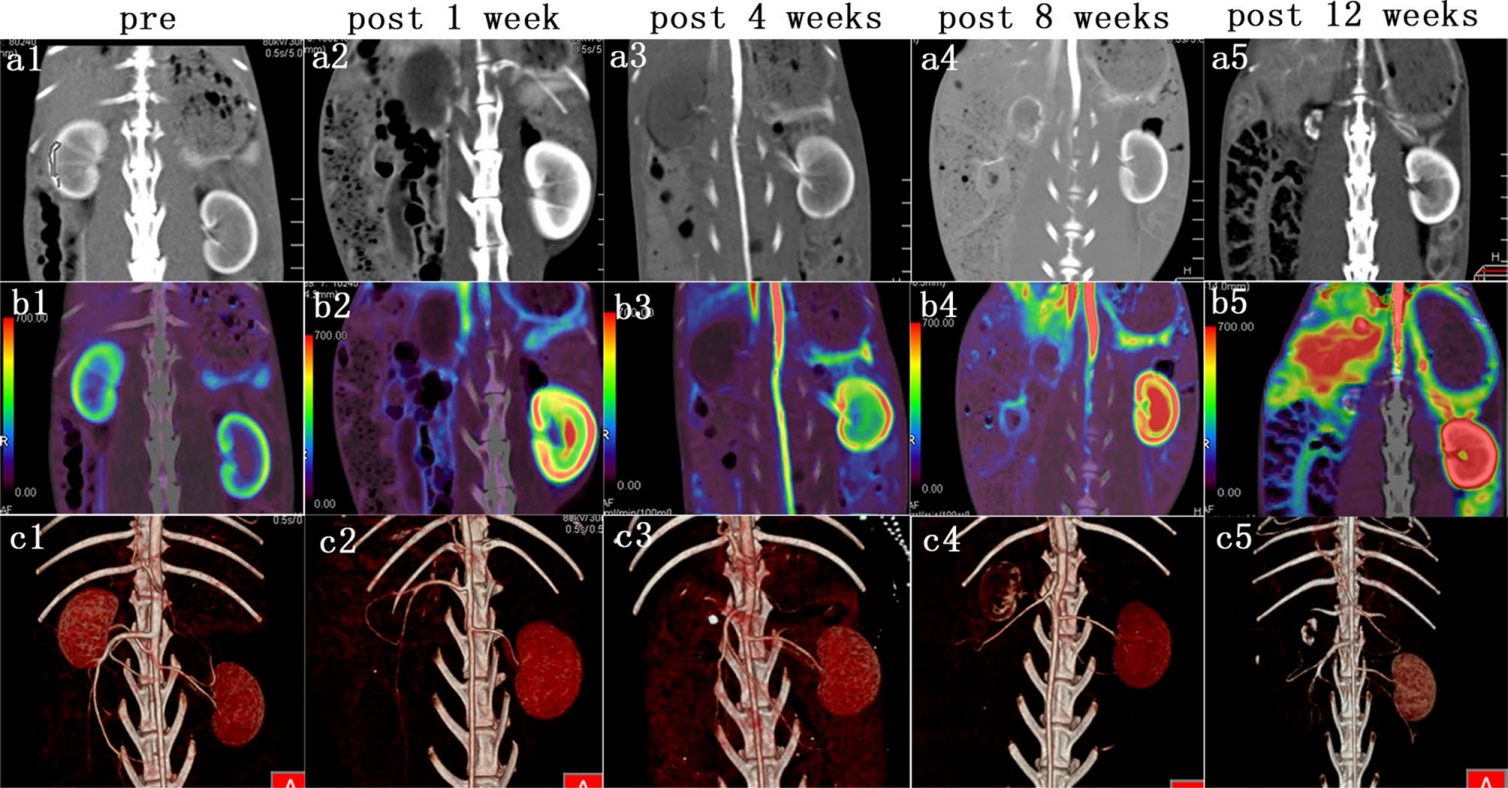
Images of CT examinations before and after embolization at various times. (**a1-5**) Enhanced images show significant shrinkage of the right kidney, with large calcifications. (**b1-5**) The perfusion maps show different colors of BF in the right kidney. (**c1-5**) The VRT images show significant calcification of the right kidney, but the left kidney and large blood vessels are clearly displayed.

**Figure 5:**
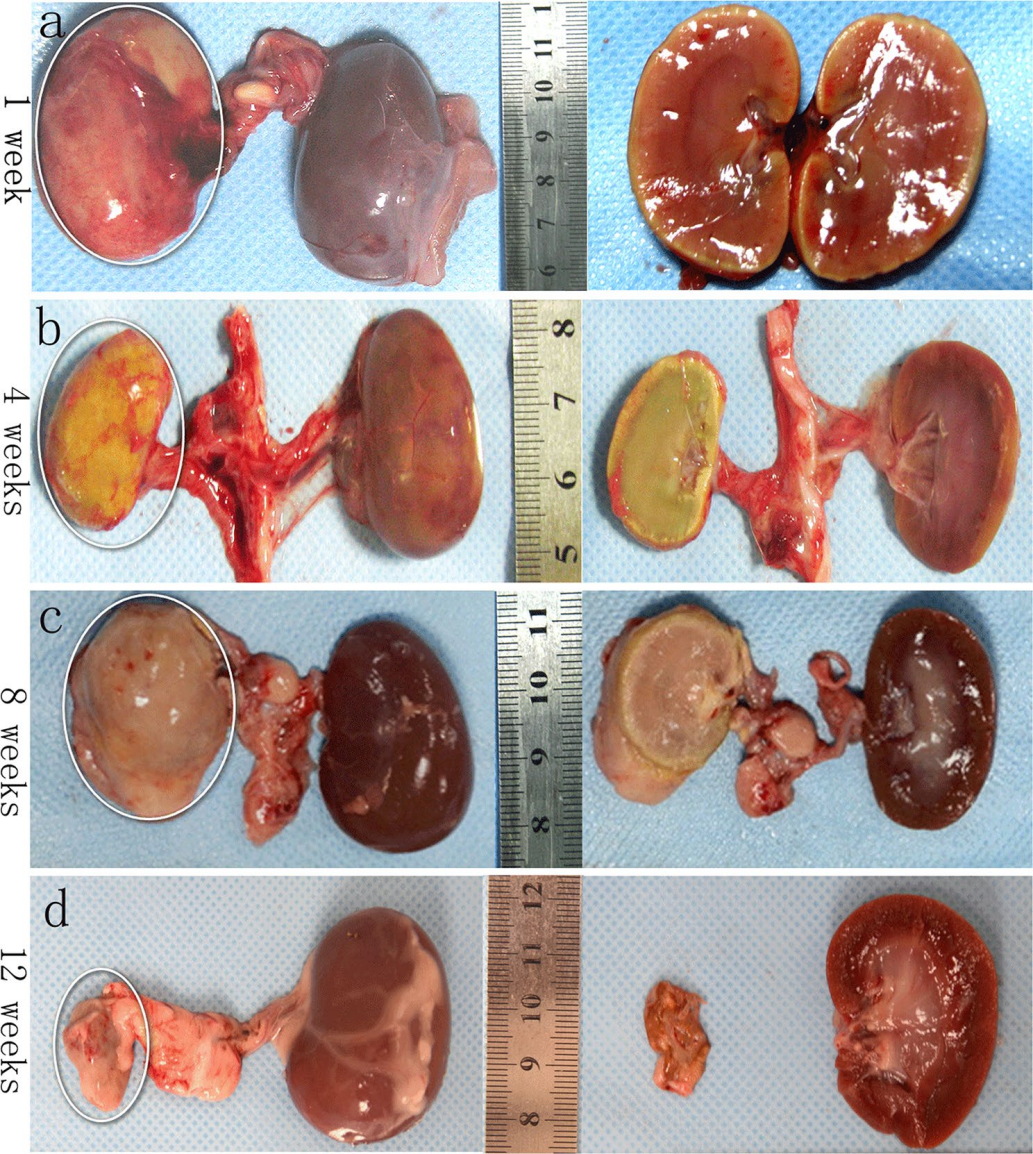
Macro-pathological pictures of the right kidneys of the rabbits after embolization at four various time points. (**a**–**d**) The images show that the right kidney gradually shrank after embolization, and the left kidney enlarged in a compensatory manner.

**Figure 6:**
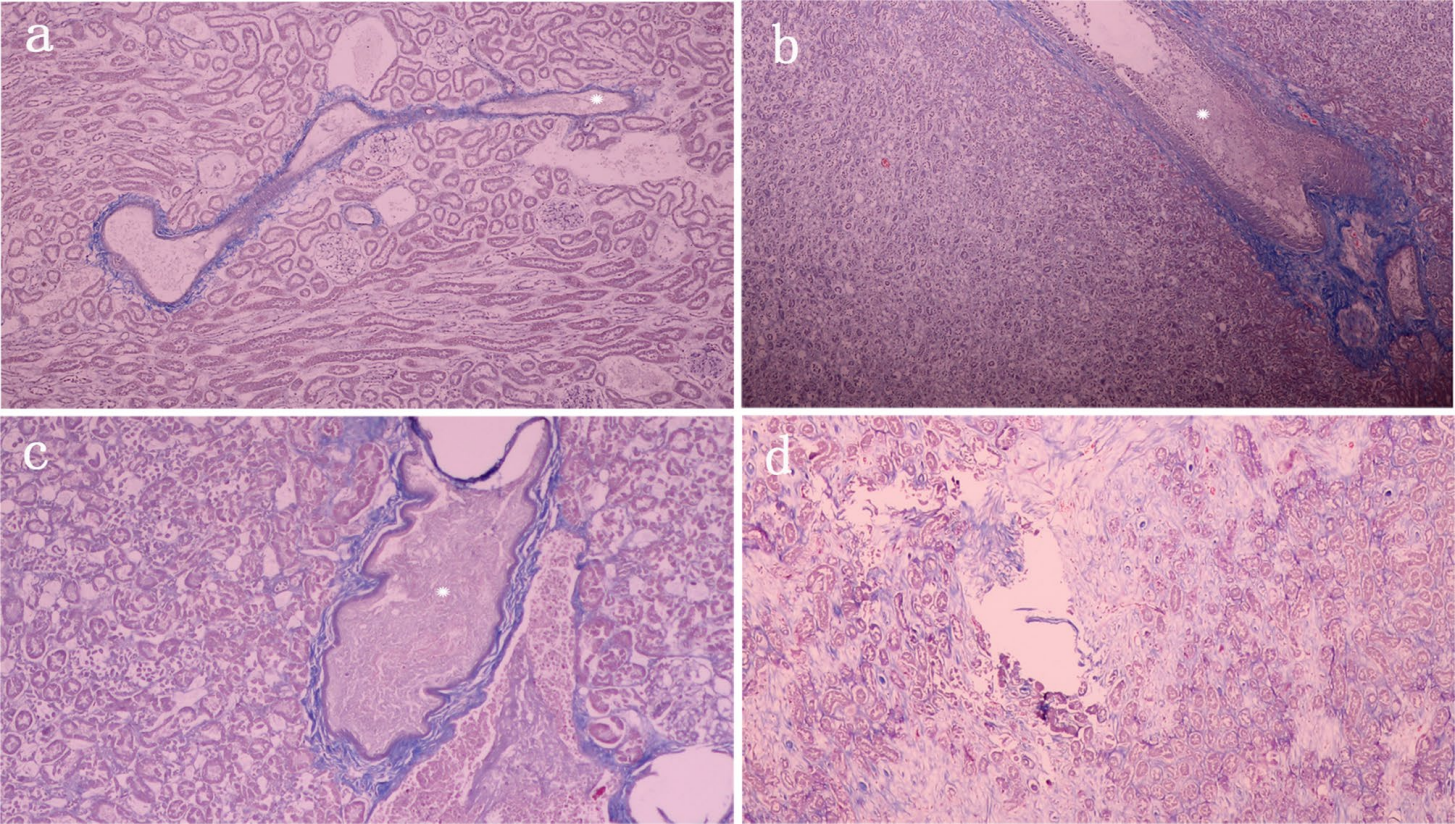
Representative images of Masson’s trichrome of the kidney at various times after embolization. (**a**) One week after embolization: a few inflammatory cells around the vascular cavity and the solid gel are visible. (**b** and **c**) Four and eight weeks after embolization: without the formation of new blood vessels, the number of fiber cells increased. (*) indicates that the PIB nanogels remained in the blood vessel. (**d**) Twelve weeks after embolization: large amount of fibrous tissue hyperplasia; solid gel encapsulation by organization. (Original magnification: 100 ×).

During the arterial embolization procedure, all levels of the renal arteries (large, small and peripheral) were embolized with PIB nanogels, indicating the PIB nanogels were well dispersed in the blood vessels. A perfusion CT examination was performed to obtain the correlation quantitative index of the paired renal volume and renal BF. These data showed that the right renal volume decreased (pre, 9278 ± 1736 mm^3^; post 1 week, 5155 ± 979 mm^3^; post 4 weeks, 3952 ± 846 mm^3^; post 8 weeks, 3226 ± 556 mm^3^; post 12 weeks, 2064 ± 507 mm^3^) and the left side increased in a compensatory manner (Fig. 3a). The right renal BF decreased from 530.81 ± 51.50 ml/min/100 ml to zero after right RAE (Fig. 3b). Significant differences were found in the renal volume (*P* < 0.0001) and BF (*P* < 0.0001) of the right kidney pre- and post-treatment during the 12 weeks. Long-term follow-up CT examination showed no BF reperfusion and revascularization after right kidney embolization (Fig. 4). Additionally, the macro-pathological pictures (Fig. 5) of RAE at four various time points (1, 4, 8, and 12 weeks) showed that the right kidney gradually shrank after embolization, and the left kidney enlarged in a compensatory manner. At the first week, the embolized kidney was yellowish white, and the renal medulla was ischemic. At the fourth week, the embolized kidney was yellow, the volume shrank, and the texture became hard, but the renal edge was yellowish white. At the eighth and twelfth weeks, the color of the right kidney was pale, the volume was smaller, and the texture was harder. Additionally, calcification of the right renal tissue was observed. Distinguishing between the cortex and medulla was difficult. The results of macroscopic examination to assess right renal volume changes were similar to those of CT perfusion imaging. These results indicated that PIB nanogels contributed to effective embolization within 12 weeks. The histological results of the right kidney indicated that the nanogels were immobilized in the renal arteries and branches throughout the entire experimental period (Fig. 6). The renal cells and tissues maintained their intact structure and morphology at 1-week post-embolization, while edema and coagulative necrosis occurred (Fig. 6a) and began to disintegrate at 4 weeks post-embolization (Fig. 6b). After embolization for 8 weeks, the pyknosis, rupture, dissolution and disappearance of renal cells indicated that PIB nanogel embolization resulted in obvious embolic gangrene, and the gel remained in the blood vessels (Fig. 6c). The structure and morphology of the renal tissues were entirely destroyed, and significant calcifications were found 12 weeks after embolization (Fig. 6d).

## Discussion

In this study, artery embolization efficacy of PIB nanogels was successfully evaluated by CT perfusion scan and histological examination before and after embolization. PIB nanogels exhibited thermo-sensitive sol–gel transition, that is, remained a liquid state at LCST and turned into a gelatinous solid as the ambient temperature increased above LCST. If their LCSTs were tailored close to physiological temperature (37 °C), ‘smart’ PIB nanogels could achieve the sol–gel transition when they entered into blood vessels and are widely used in blood vessel embolic materials^11,14^, drug delivery^20–22^, precise cancer therapy^23,24^, tissue engineering^25^, and cell sheet engineering^26,27^. Most of the studies focused on their phase transition behavior theory, drug delivery and promising biomedical applications. However, little has been reported on the clinical transition of the smart polymeric materials.

The ideal vascular embolic material has the following characteristics: favorable dispersibility, radiopacity, biocompatibility and embolism permanency^10,11^. In previous studies, PIB nanogels have been confirmed to be non-adhesive, controllable, and low inflammatory reactions of the blood vessels^12^. Transcatheter arterial embolization (TAE) treatment is highly dependent on the dispersion and integration of the embolization material. Temperature-sensitive PIB nanogels remain liquid at a temperature lower than their LCST (35 °C) and have good flowability in fine catheters. When they enter in vivo, the body temperature is higher than their LCST (35 °C), PIB nanogels change from a liquid state to a gelatinous solid in response to ambient temperature, and in situ gelate in blood vessels, matching well the shape and size of the targeted blood vessels. PIB nanogels achieved effective RAE that was uniform and persistent because the embolic agent was stable in the embolized vessel throughout the entire 12 weeks, with no microvascular formation in the embolized kidney (Fig. 6). This phenomenon led to a significant drop in the right kidney volume and BF after embolization (Fig. 3). Atrophy and calcification were observed in the right kidneys (Fig. 4). Because of the different proficiencies of the operators, the segment of the renal artery embolism might be slightly different. After embolization of the peripheral vessels, the catheter was slightly retracted to embolize the proximal renal artery. The segment of renal artery embolization varied with the distance of catheter retraction. When the catheter was retracted a shorter distance, the distal renal artery was embolized, resulting in segmental embolization of the renal artery and making the renal artery appear relatively intact (Fig. 5b); however, the kidney was obviously affected. Additionally, the PIB nanogels remained in the blood vessel during the 12-week follow-up based on pathologic examination (Fig. 6), confirming the embolization effect of the PIB nanogels. Regarding the nanogel dose, our previous study had shown that various levels of renal arteries could be embolized by adjusting the nanogel injection dosages, and a dose of 2 ml could embolize all levels of renal arteries (including peripheral, small and large arteries)^16^. Zhao^12^ studied the dispersion of the gel according to different injection rates; however, the long-term effects of the treatment were not observed by the imaging methods and remain unknown. Additionally, DSA is a traumatic examination that may cause various complications^15,28–30^, increasing the risk of death in experimental animals. In this study, we observed the dispersion of PIB in the right kidney and evaluated both the long-term effect of treatment by CT, macroscopic and histological examination in a 12-week follow-up examination.

CT perfusion is a functional imaging technique^17,31,32^ that can noninvasively evaluate the blood supply and kinetics, reflecting physiological function changes. An important use of CT perfusion is the evaluation of cerebral ischemia, which can provide important evidence to support the treatment choice of patients with ischemic stroke^33,34^. Recently, studies have indicated the potential of CT perfusion to predict angiogenesis and treatment efficacy in multiple cancers, such as hepatocellular carcinoma^17^, renal cell carcinoma^35^, lung cancer^31^, and gastric cancer^32^, among others. In this study, we evaluated the embolization effect of the right renal artery using CT perfusion, a major advantage of which is its non-invasive nature. Additionally, this method can depict the morphologic characteristics of the kidney and provide additional quantitative factors to reflect the embolization effect. During follow-up, the right kidney volume decreased gradually after embolization, while the volume of the left kidney increased in a compensatory manner after the operation and then decreased slightly. After embolizing the right renal artery, the right renal BF decreased significantly to 0, and the left renal BF increased in a compensatory manner. The lack of vascular enhancement reflects necrosis and is regarded as successful treatment^36^, demonstrating that the gelling agent has excellent permanency. Macroscopic examination of renal tissue at post-mortem verified the results obtained using CT perfusion; thus, evaluating the embolic effect is feasible by CT perfusion.

The current study has some limitations. First, because of the different proficiency of operators, the position of renal artery embolism was slightly different. Second, the number of rabbit samples was limited, and further studies are warranted. Studies regarding these properties of our new gel are now ongoing.

## Conclusion

The effect of embolization on PIB was adequate, with good dispersion and permanency. The nanogel can reach peripheral blood vessels and remain in them for a long time. Thus, this gel is a promising blood vessel embolic material and can be used in vivo. CT perfusion can be used to non-invasively and quantitatively evaluate the embolization effect of temperature-sensitive nanogels.
